# The risk of development and progression of diabetic retinopathy in a group of ethnically diverse pregnant women with diabetes attending three regional Diabetic Eye Screening Programs in the UK

**DOI:** 10.1038/s41433-023-02655-0

**Published:** 2023-07-07

**Authors:** Kirsty Clarke, Laura Webster, Susanne Althauser, John Anderson, Irene Stratton, Anna Brackenridge, Samantha S. Mann

**Affiliations:** 1 Guy’s and St Thomas’ Hospital NHS Trust, London, England; 2 Imperial College London, London, England; 3 South East London Diabetic Eye Screening Programme, London, England; 4 North Central London Diabetic Eye Screening Programme, London, England; 5 North East London Diabetic Eye Screening Service, Ilford, England; 6 Gloucestershire Hospitals NHS Foundation Trust, Gloucester, England

**Keywords:** Epidemiology, Epidemiology

## Abstract

**Background/Objectives:**

Currently, all pregnant women with diabetes are asked to attend screening at least twice during pregnancy, even if no retinopathy is detected in early pregnancy. We hypothesise that for women with no diabetic retinopathy in early pregnancy, the frequency of retinal screening may be safely reduced.

**Subjects/Methods:**

In this retrospective cohort study, data for 4718 pregnant women attending one of three UK Diabetic Eye Screening (DES) Programmes between July 2011 and October 2019 was extracted. The women’s UK DES grades at 13 weeks gestation (early pregnancy) and 28 weeks gestation (late pregnancy) were recorded. Descriptive statistics were used to report baseline data. Ordered logistic regression was used to control for covariates, such as age, ethnicity, diabetes duration, and diabetes type.

**Results:**

Of the women with grades recorded for both early and late pregnancy, a total of 3085 (65.39%) women had no retinopathy in early pregnancy, and 2306 (74.7%) of these women did not develop any retinopathy by 28 weeks. The number of women without retinopathy in early pregnancy who developed referable retinopathy was 14 (0.45%), none of whom required treatment. Diabetic Retinopathy in early pregnancy remained a significant predictor of DES grade in late pregnancy when covariates of Age, Ethnicity, and Diabetes Type were controlled for (*P* < 0.001).

**Conclusions:**

In summary, this study has demonstrated that the burden of managing diabetes for pregnant mothers may be safely reduced by limiting the number of diabetic eye screening appointments in women who have no retinal changes in early pregnancy. Screening of women with retinopathy in early pregnancy should continue in line with current UK guidance.

## Introduction

The global prevalence of diabetes is set to double over the next 20 years [1]. However, the global incidence and prevalence of sight-threatening diabetic retinopathy have decreased, such that, in the United Kingdom, diabetic retinopathy is no longer the leading cause of blindness in the working-age population [2]. This trend likely reflects the cumulative impact of earlier and more effective ophthalmologic interventions, screening, improved glycaemic control, and early risk factor modification [3].

The presence of diabetic retinopathy in early pregnancy is an important predictor for the development of sight-threatening (referable) diabetic eye disease during pregnancy (UK Diabetic Eye Grading Criteria) [4, 5]. Women enrolled in the Diabetes In Early Pregnancy study (DIEP) who had no retinopathy at conception had a 10.3% risk of progression. In comparison, those with moderate non-proliferative retinopathy carried a 58% risk of progression (>2 UK Diabetic Eye Screening grades). No women with no retinopathy or only microaneurysms at conception developed proliferative disease. Diabetes duration, glycaemic control, age, and gravidity were also identified as independent risk factors for retinopathy progression [4]. Similarly, In a cohort of 348 pregnant women attending routine diabetic eye screening in Denmark, the majority of women did not have retinopathy in early pregnancy, none of whom developed sight-threatening retinopathy so long as appropriate glycaemic control was maintained [6].

Screening guidelines in the US and Denmark have already pivoted in response to this evidence and now advise that retinopathy screening beyond early pregnancy may be safely omitted in women without retinopathy and with good glycaemic control in early pregnancy [6]. However, the UK’s National DESP has yet to adjust. Instead, the recommended photo-screening frequency remains the same as when the programme was first devised in 2003 [7], potentially over-burdening an already overwhelmed system. It is also important to note that excess screening visits also threaten the emotional well-being of pregnant women, who already face challenges associated with managing diabetes and pregnancy.

Currently, all pregnant women with diabetes are asked to attend DESP at least twice during pregnancy, once before 13 weeks (early pregnancy) and once at 28 weeks (late pregnancy) if no retinopathy is detected in early pregnancy and three times if any retinopathy is detected at the first screen [7]. However, as the NHS faces an ever-increasing demand for its services, there is both a fiscal and ethical responsibility to ensure the frequency of photo screening is reviewed in line with current evidence. We hypothesise that for women with no diabetic retinopathy (R0M0) in early pregnancy, the frequency of retinal screening could be safely reduced to minimise the burden of health care visits. Thus, this study aims to use pooled data from three diabetic screening centres in North East, South East, and North Central London to evaluate the following three outcomes, ensuring that covariates such as diabetes duration, ethnicity, diabetes type, and age at conception are controlled for.

1. The percentage of women with no retinopathy in early pregnancy (R0M0 before 13 weeks gestation) who did not develop any retinopathy during pregnancy.
2. The percentage of women with no retinopathy in early pregnancy (R0M0 before 13 weeks gestation) who developed early non-referable retinopathy (R1M0, mild non-proliferative) during pregnancy.
3. The percentage of women with no retinopathy in early pregnancy (R0M0 before 13 weeks gestation) who developed referable retinopathy (moderate to severe non-proliferative, proliferative retinopathy or maculopathy) during pregnancy (R1M1 or R2M0 or above).

## Subjects and methods

**Fig. 1:**
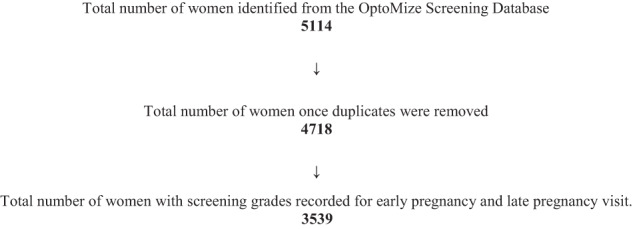
A flow diagram depicting the participant selection and inclusion process. As shown in the flow diagram, 5114 women were initially identified from the OptoMize Screening Database, and 3539 (69%) of these women met the study inclusion criteria.

In this retrospective cohort study, screening data for all pregnant women attending DESP in North East, South East, and North Central London between July 2011 and October 2019 were identified from the OptoMize Screening Database. Data for those who were known to have had at least one pregnancy were extracted and pseudo-anonymized. The women’s diabetic eye service screening grades in early and late pregnancy were derived from fundal photo images taken before 13 weeks gestation and at 28 weeks gestation, respectively. Fundal photographs were taken as part of the UK Diabetic Eye Screening Programme, where two-field (disc- and macula-centred) 45° colour fundus photographs using pupil dilation (mydriasis) are routinely taken. Fundal Images are then assessed by human graders for the presence of both retinopathy and maculopathy. The worst eye grade from each screening event was used for the analysis. A total of 5114 women were identified from the OptoMize database, and 4718 remained once duplicates were removed. The flow diagram in Fig. 1 describes the process of participant selection and inclusion. Data pertaining to age, ethnicity, diabetes duration, and diabetes type were collected for the remaining 4718 women and are displayed in Table 1. Unfortunately, data for other risk factors, such as blood pressure and HbA1c, were not available.

**Table 1:** Summary of Study Participant Characteristics.

		Women Meeting Study Inclusion Criteria (*n* = 4718)
Median age at conception (IQR)		34 (8)
Median diabetes duration, months (IQR)		89 (96)
Diabetes Type (%)	Type 1 Diabetes	1094/4718 (23%)
	Type 2 Diabetes	3239/4718 (69%)
	MODY/Other	93/4718 (2%)
	Unknown	291/4718 (6%)
	**Total**	**4718 (100%)**
Ethnicity (%)	African	988/4718 (21%)
	Any other Asian background	243/4718 (5%)
	Any other Ethnic Group	166/4718 (4%)
	Any other Mixed background	110/4718 (2%)
	Bangladeshi	884/4718 (19%)
	White/ White other	1271/4718 (27%)
	Indian	299/4718 (6%)
	Pakistani	229/4718 (5%)
	Unknown	528/4718 (11%)
	**Total**	**4718 (100%)**

Retinopathy grades were defined using the criteria of the UK National Screening Committee, as shown in Table 2, and the earliest pregnancy on record was used for each woman. Therefore, non-referable retinopathy refers to screening grades R0M0 (no diabetic retinopathy or maculopathy) or R1M0 (mild non-proliferative diabetic retinopathy). Referable retinopathy includes screening grades R1M1 (mild non-proliferative diabetic retinopathy with maculopathy), R2M0 (moderate to severe non-proliferative diabetic retinopathy only), R2M1 (moderate to severe non-proliferative diabetic retinopathy with maculopathy), R3M0 (proliferative retinopathy with no maculopathy), and R3M1 (proliferative diabetic retinopathy with maculopathy).

**Table 2:** Summary of participant characteristics for women who progressed from R0M0 to R1M1 or worse during pregnancy.

Median Age at Conception (IQR)		34 (6)
Median Diabetes Duration, months (IQR)		88 (90)
Diabetes Type (%)	Type 1 Diabetes	3/14 (21%)
	Type 2 Diabetes	11/14 (79%)
	**Total**	**14 (100%)**
Ethnicity (%)	African	8/14 (57%)
	Any other Mixed background	2/14 (14%)
	Bangladeshi	2/14 (14%)
	White/White Other	2/14 (14%)
	**Total**	**14 (100%)**
Retinopathy grade at follow-up (%)	R1M1	13/14 (93%)
	R2M1	1/14 (7%)
	**Total**	**14 (100%)**

Frequency tables, medians, and interquartile ranges were used to report baseline data. As all assumptions were met, including proportional odds and exclusion of multicollinearity, ordered logistic regression was used to control for covariates, such as age, ethnicity, diabetes duration, and diabetes type. A two-sided *p*-value < 0.01 was considered statistically significant. All statistical analysis was done using SPSS Statistics Software IBM® Version 27.

## Results

**Fig. 2:**
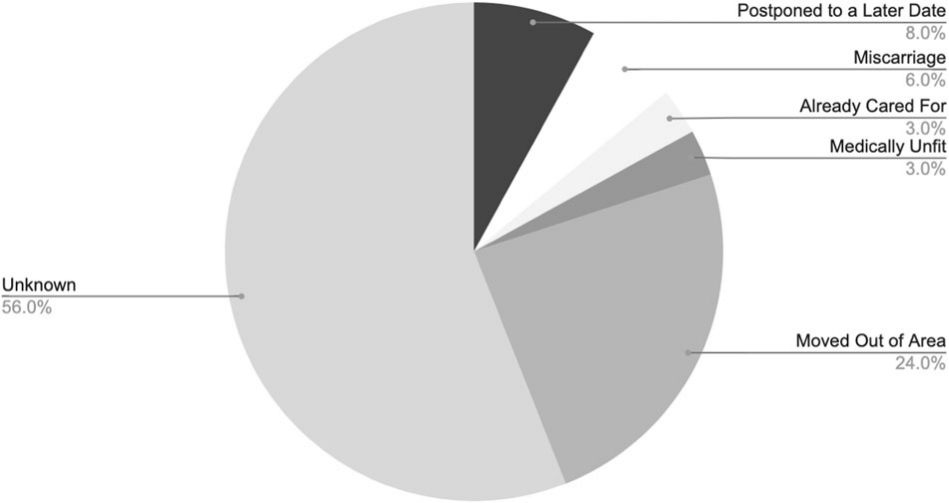
Reasons given for patients missing their second screening appointment.

A total of 4718 women were included in the study, 1094 (23%) with type 1 diabetes and 3239 (69%) with type 2. For 3539 women, both an early pregnancy and late pregnancy screening grade could be identified. The sample size adequately exceeded the minimum number of measurements (3037) needed to provide a confidence level of 95% that the real value is within ±1% of the measured value. For most women with only one screening grade, it was the late pregnancy screening grade which was absent. Reasons for women not attending their second screening visit are detailed in Fig. 2.

A summary of the screening grades recorded in early and late pregnancy and rates of progression are shown in Table 3. Of the women who had grades recorded for both early and late pregnancy screening visits, a total of 3085 (65.39%) women had no retinopathy in early pregnancy (R0M0 before 13 weeks), and the percentage of these women who did not develop any retinopathy during pregnancy was 2306 (74.7%). The number of women who had no retinopathy in early pregnancy (R0M0 before 13 weeks) who developed early non-referable retinopathy (R1M0, mild non-proliferative) was 250 (8.1%). No women with no retinopathy in early pregnancy (R0M0 before 13 weeks) developed R3 grade (proliferative) retinopathy. As shown in Table 2, only 14 (0.45%) developed referable retinopathy or maculopathy (R1M1 and above), and after checking their hospital record, it was confirmed that none of these patients required any further treatment upon referral to ophthalmology.

**Table 3:** Screening Grades in early and late pregnancy in women who attended both screening appointments based on UK Diabetic Eye Screening Programme Criteria (shaded area represents referable maculopathy or retinopathy).

		Retinopathy grade in late pregnancy (28 weeks gestation)									
		R0M0	R1M0	R1M1	R2M0	R2M1	R3M0	R3M1	Ungradable*	Did not attend	Total
Retinopathy grade in early pregnancy (<13 weeks gestation)	R0M0	2306	250	13	0	1	0	0	8	507	3085
	R1M0	173	575	53	16	5	2	0	2	138	964
	R1M1	5	29	41	1	7	1	1	0	79	164
	R2M0	0	5	2	9	6	2	1	1	12	38
	R2M1	0	0	3	2	7	2	3	0	20	37
	R3M0	0	1	1	1	0	4	1	0	13	21
	R3M1	1	0	1	1	2	3	3	0	22	33
	Ungradable*	10	5	0	0	0	2	0	0	5	22
	Did not attend	3	1	1	0	0	0	0	0	349	354
	Total	2498	866	115	30	28	16	9	11	1145	4718

*‘Ungradable’ eyes included those cataracts or other ocular pathology which made image interpretation unreliable. All ungradable eyes are seen later in the slit-lamp retina clinic.

Ordered logistic regression (see Supplemental Material) revealed that no retinopathy in early pregnancy (R0M0 before 13 weeks) remained a significant predictor of Diabetic Eye Screening grade in late pregnancy when the covariates of Age, Ethnicity, and Diabetes Type were controlled for (*P* < 0.001). All grades higher than R1M0 predicted a worsening of the follow-up grade (*P* < 0.005). Duration of diabetes predicted worsening of diabetic eye screening grade by a factor of 0.004, to that of the early pregnancy grade (*P* < 0.001). Older age predicted worsening of diabetic eye screening grade by a factor of 0.02, to that of the early pregnancy grade (*P* < 0.001). Type 1 diabetes predicted worsening of diabetic eye screening grade by a factor of 0.450, to that of the early pregnancy grade (*P* < 0.001). No relationship between other diabetes types and late pregnancy screening grade was found. Ethnicity did not predict retinopathy grade in late pregnancy, but 10 out of 14 of those that progressed to a referable grade were from an ethnic minority background, and 8 were of African ethnicity.

## Discussion

This retrospective cohort study analysed data from 4718 women attending one of three Diabetes Screening programmes in South East, North East and North Central London. To our knowledge, this is the largest study to report the risk of diabetic retinopathy progression and development in relation to early pregnancy screening grade in the UK screening population. In this study, no women without retinopathy in early pregnancy developed R3 disease (proliferative retinopathy). The risk of developing the referable disease was also very low (0.45%), altogether indicating that repeat screening in late pregnancy may be unnecessary for these women, especially if their other risk factors for progression are controlled. Our findings are in keeping with the decreasing incidence and prevalence of sight-threatening retinopathy reported globally, a trend which has already led to a revision of screening guidance in the US and Europe, such that routine diabetic eye screening beyond early pregnancy in women without retinopathy and with good glycaemic control is no longer advised [6]. As most women attending the three included diabetic screening centres in this study did not have retinopathy in early pregnancy (65% R0M0), our data suggest a large reduction in the screening caseload could be achieved if the UK implemented similar changes.

Moreover, of the women in our study with pre-existing diabetic eye disease in early pregnancy, the risk of their disease worsening remained significant (9.83%), even in those with mild non-referable (R1M0) eye disease in early pregnancy (8.1%). Similarly, in the DIEP prospective cohort study, which followed up 155 women peri-conceptional period to 1 month postpartum, the risk of progression to a grade of R3 during pregnancy for women with R0 or mild R1 at baseline was low, at 0.4% [4]. The progression of ≥2 Diabetic Eye Screening grades occurred in 10.3% of women with R0 at baseline, 18.8% with mild non-proliferative diabetic retinopathy (NPDR) at baseline, and 54.8% with moderate NPDR or worse at baseline [4]. Additionally, in an uncontrolled prospective study of 179 women with type-1 diabetes conducted by Temple et al., the overall progression of sight-threatening diabetic retinopathy was found to be significantly more common in women with moderate or severe background diabetic retinopathy at booking compared with women with minimal or no retinopathy at booking (30% vs 3.7%, *P* = 0.01) [8]. Based on this evidence, regular screening of women with referable and mild non-referable retinal changes in early pregnancy remains advisable.

Furthermore, the risk of retinopathy progressing in the postpartum period remains an important consideration [9]. While we do not have access to postpartum screening data for the women in this study, none of the women in our study who had no retinopathy in early pregnancy and later developed referable disease required any treatment during the postpartum period. In addition, while there is evidence to suggest that retinopathy may progress after pregnancy, few studies have explored the extent of such progression [10, 11]. For example, the retrospective analysis conducted by Lauszus et al. reports retinopathy progression for 19 women postpartum compared to 7 during pregnancy. However, it is unclear whether these women’s retinopathy progressed to a referable grade, and importantly, no women whose retinopathy progressed after pregnancy required intervention [10]. There is also strong evidence to suggest that regression of retinopathy is more common in the postpartum period [9], with a recent retrospective study of 499 women reporting regression and progression rates of 9.3% and 4.1%, respectively [11].

Notably, in this study, the absence of retinopathy in early pregnancy remained a significant predictor of diabetic eye screening grade at the end of pregnancy, even when covariates known to influence progression, such as age, diabetes type, and ethnicity, were controlled for. Contrary to what might have been expected [12], African, African Caribbean and South Asian ethnicities did not significantly predict retinopathy progression. However, 10 (71%) of the 14 patients with no retinopathy who developed referable retinopathy or maculopathy during pregnancy were from high-risk ethnic groups, compared to 2643 (56%) in the baseline sample.

Also, unlike other studies [6, 8, 10, 11], our study cohort consisted mainly of women with type 2 diabetes (69%) rather than type 1, potentially reflecting the increasing prevalence of type 2 diabetes in women of childbearing age in the UK. Notably, a recent meta-analysis has suggested that even though the prevalence of retinopathy in pregnancy is higher in patients with type-1 diabetes, the risk of retinopathy progression during pregnancy did not significantly differ between types [13]. Nonetheless, of the 14 women who developed referable retinopathy in our study, the majority had type-2 diabetes (79%), and all developed maculopathy. Importantly, while the high maculopathy rate in this study could be explained by the increased proportion of women with type 2 diabetes in our cohort [14], improved detection methods, such as OCT scanning, are also likely to have played a role. As stated above, most of our cohort developing new maculopathy were from high-risk ethnic groups (71%), and the DRIVE UK study has demonstrated that those of Afro-Caribbean origin are more likely to develop maculopathy than retinopathy [12]. Thus, our findings emphasise the need to closely monitor those with retinopathy in their first trimester who possess underlying risk factors for progression, regardless of their diabetes type.

Regrettably, data pertaining to several known risk factors for retinopathy progression, such as blood pressure, pre-eclampsia, and blood sugar control, are not routinely collected as part of eye screening in the UK and therefore were not included in our data analysis [15]. While hypertension is known to be a powerful risk factor for retinopathy progression [15], both a recent retrospective [11] and a prospective study of pregnant women with insulin-dependent diabetes [10] failed to correlate arterial hypertension with any increased risk of progression in women with tightly controlled blood sugar levels. Similarly, in Pappot et al.’s retrospective cohort analysis of 348 women with diabetes, no women without retinopathy and HbA1c < 53 mmol/mol (7.0%) in early pregnancy developed sight-threatening retinopathy and 94% (165/175) did not develop any retinopathy [6]. However, one woman with no retinopathy but an HbA1c > 53 mmol/mol (7.0%) developed clinically significant macular oedema [6]. It could, therefore, be hypothesised that variation in glycaemic control could account for the small fraction of R0M0 patients whose retinopathy progressed in this study. Furthermore, it is known that if any deterioration occurs in women with poor glycaemic control, it will most likely arise in the first trimester (before 13 weeks) and is likely, therefore, to be detected at the early screening visit [16]. Overall, it may be advisable that pregnant women with particularly poor glycaemic control should continue to be offered screening at an increased frequency.

A key strength of this study was the evaluation of both diabetic retinopathy and maculopathy in a large, ethnically diverse cohort, including both women with type 1 and type 2 diabetes. The external validity of the study is, however, limited to London, and findings may not be representative of other parts of the UK. Though, as our study population represents some of the UKs most ethnically diverse DESPs and the risk of retinopathy is known to be higher in people of African/Afro-Caribbean ethnicity compared to those who are South Asian or White, we would predict the risk of retinopathy progression to be even lower in most other UK DESPs. This considered, screening data from London does have the disadvantage that many patients initially screened in London are later referred to their local service or moved out of the area, which contributed to a higher dropout rate at the second screen in our cohort (see Fig. 2). While the authors accounted for this in the data analysis, and the participant dropout rate was comparable to attrition rates in other studies (7.84%) [6, 8], it is possible that this skewed the results.

In summary, this study has demonstrated that the burden of managing diabetes for pregnant mothers may be safely reduced by limiting the number of diabetic eye screening appointments in women who have no retinal changes (R0M0) in early pregnancy. More research is needed to evaluate why a small percentage of women with no diabetic eye disease seem to develop referable retinal changes during their pregnancy, although, in this study, none of these developed proliferative retinopathy and none required any treatment for retinopathy or maculopathy once referred to the eye clinic. Further evaluation of the role of glycaemic control, blood pressure control, and the risk of retinopathy progression after delivery in such patients may be beneficial. It may be that those with particularly high HbA1c, or hypertension at the start of pregnancy would continue to be offered a higher frequency of screening. Overall, a greater understanding of risk factors for retinopathy progression in pregnancy may enable the development of individualised retinal examinations, similar to those forecasted for diabetic patients attending routine diabetic eye screening in the UK, where a longer follow-up of two years before re-screening has been suggested in those with no retinopathy (R0M0) who are believed to be at a lower risk of diabetic eye disease progression [11, 12].

This retrospective cohort study analysing data from over 4700 women attending both early and late pregnancy diabetic eye screening appointments in the UK has revealed that the risk of women with no retinopathy (R0M0) in early pregnancy developing referable retinopathy during pregnancy is minimal, and those that do progress to referrable retinopathy are unlikely to require any treatment. Therefore, the authors suggest that current guidance recommending further screening in late pregnancy for these women is reviewed, especially if no other risk factors for retinopathy progression are present. For women with pre-existing diabetic retinopathy in early pregnancy, the risk of disease progression during pregnancy remains significant, and screening of these women should continue in line with current UK guidance.

## Summary

### What was known before:

- The UK Diabetic Eye Screening programme currently screens pregnant women at an increased frequency due to the risk of developing sight-threatening retinopathy so they can receive appropriate treatment if needed. In recent years, risk factor modification, including improved glycaemic control, has led to a decline in the incidence of sight-threatening retinopathy. Evidence from abroad has emerged suggesting that this also applies to pregnant women with no retinopathy at conception/ early pregnancy who have a minimal risk of developing sight-threatening retinopathy. To reduce the morbidity of unnecessary screening visits for women with diabetes, National diabetic eye screening programmes in the US and Denmark have safely reduced the screening interval in women with no retinopathy, considered to be at low risk of developing sight-threatening retinopathy

### What this study adds:

- Findings from this retrospective cohort study of 4718 pregnant women, enrolled in three UK regional Diabetic Eye Screening programmes, suggest that the risk of women with no retinopathy in early pregnancy developing referable retinopathy during pregnancy is minimal, and those that do progress are unlikely to require treatment. For women with pre-existing diabetic retinal changes in early pregnancy, the risk of disease progression during pregnancy remains significant, and screening of these women should continue in line with current UK guidance. The burden of managing diabetes for pregnant mothers may be safely reduced by limiting the number of diabetic eye screening appointments, even in areas with high ethnic diversity, as part of the UK diabetic eye screening programme in women with no diabetic retinopathy changes documented in early pregnancy.

### Supplementary information

Supplementary Infomation

## Data Availability

Derived data supporting the findings of this study are available from the corresponding author (KC) upon reasonable request.
