# Supplementary material for: The risk of development and progression of diabetic retinopathy in a group of ethnically diverse pregnant women with diabetes attending three regional Diabetic Eye Screening Programs in the UK
Source: Eye (Lond). 2023 Jul 7;38(1):179–84. doi: 10.1038/s41433-023-02655-0 (PMC10764873; doi:10.1038/s41433-023-02655-0)
Supplement: Supplementary file 1 — Supplementary Infomation [file 41433_2023_2655_MOESM1_ESM.docx]

| **Supplementary Figure 1- Ordered Logistic Regression results** | | | | | | |  | |
| --- | --- | --- | --- | --- | --- | --- | --- | --- |
|  | | | | |  | |  | |
|  |  | Estimate | Std. Error | Wald | Sig. | 95% Confidence Interval | |  |
|  |  |  |  |  |  | Lower limit | Upper limit |  |
| *Diabetes Duration* |  | 0.004 | 0.000 | 71.83 | <.001 | 0.003011 | 0.005 |  |
| *Age* |  | 0.02 | 0.006 | 10.63 | 0.001 | -0.033 | -0.008 |  |
| *Ethnicity* |  | -0.309 | 0.338 | 0.838 | 0.36 | -0.972 | 0.353 |  |
| *Diabetes Type* | MODY | -0.311 | 0.581 | 0.286 | 0.593 | -1.449 | 0.828 |  |
|  | Not Specified | -0.354 | 0.274 | 1.67 | 0.196 | -0.89 | 0.183 |  |
|  | Type 2 | -0.48 | 1.482 | 0.105 | 0.746 | -3.384 | 2.424 |  |
|  | Other | 0.248 | 0.311 | 0.635 | 0.425 | -0.361 | 0.857 |  |
|  | **Type 1** | **0.45** | **0.111** | **16.41** | **<.001** | **0.232** | **0.668** |  |
| *Early screening grade (<13 weeks gestation)* | **Not Specified** | **-1.222** | **0.562** | **4.722** | **0.03** | **-2.324** | **-0.12** |  |
|  | **R0M0** | **-1.634** | **0.548** | **8.877** | **0.003** | **-2.708** | **-0.559** |  |
|  | R1M0 | 0.444 | 0.549 | 0.654 | 0.419 | -0.632 | 1.52 |  |
|  | **R1M1** | **1.643** | **0.574** | **8.197** | **0.004** | **0.518** | **2.768** |  |
|  | **R2M0** | **1.919** | **0.642** | **8.922** | **0.003** | **0.66** | **3.178** |  |
|  | **R2M1** | **2.861** | **0.643** | **19.78** | **<.001** | **1.6** | **4.122** |  |
|  | **R3AM0** | **3.676** | **0.838** | **19.26** | **<.001** | **2.034** | **5.318** |  |
|  | **R3AM1** | **3.297** | **0.866** | **14.49** | **<.001** | **1.599** | **4.995** |  |
|  | **R3M0** | **4.881** | **1.407** | **12.03** | **<.001** | **2.123** | **7.639** |  |
|  | **R3SM1** | **5.209** | **1.932** | **7.272** | **0.007** | **1.423** | **8.995** |  |

Supplementary Table NHS Diabetic Eye Screening Programme Feature Based Grading Classification

| R0 | None | No DR |  |
| --- | --- | --- | --- |
|  |  |  |  |
| R1 | Background | microaneurysms |  |
|  |  | retinal haemorrhage(s) |  |
|  |  | venous loop |  |
|  |  | any exudate in the presence of other features of DR |  |
|  |  | any number of cotton wool spots (CWS) in the presence of other features of DR |  |
| **R2** | **Pre-proliferative** | venous beading |  |
|  |  | multiple blot haemorrhages |  |
|  |  | intraretinal microvascular abnormality (IRMA) |  |
| **R3** | **Proliferative** |  |  |
|  |  | new vessels on disc (NVD) | R3a (Active Proliferative Retinopathy) |
|  |  | new vessels elsewhere (NVE) |  |
|  |  | New pre-retinal or vitreous haemorrhage |  |
| **M** |  | **Maculopathy** |  |
| M0 |  | No maculopathy | Absence of any M1 features |
| M1 |  | exudate, haemorrhage or microaneurysm within 1 disc diameter (DD) of the centre of the fovea |  |
| **U** |  | **Ungradable** |  |
